# Laryngeal Frame Involvement as The First Sign of Wegener’s Granulomatosis

**DOI:** 10.22038/ijorl.2024.81617.3746

**Published:** 2025

**Authors:** Davide Burrascano, Barbara Verro, Gaetano Ottoveggio, Ada Maria Florena, Carmelo Saraniti

**Affiliations:** 1 *Unit of Otorhinolaryngology, Department of Biomedicine, Neuroscience and Advanced Diagnostic, University of Palermo, 90127 Palermo, Italy.*; 2 *Unit of Anesthesia, Intensive Care, and Emergency, Department of Precision Medicine in Medical, Surgical and Critical Care (Me.Pre.C.C.), University of Palermo, Italy.*; 3 * Department of Sciences for Promotion of Health and Mother and Child Care, Anatomic Pathology, University of Palermo, Italy.*

**Keywords:** Granulomatosis, Wegener, ANCA, Antineutrophil Cytoplasmic Antibodies, larynx

## Abstract

**Introduction:**

Granulomatosis with Polyangiitis (GPA), also known as Wegener’s Granulomatosis, is an ANCA-associated vasculitis that primarily affects small vessels, leading to necrotizing granulomatous reactions in the airways and small vessels. The etiology remains uncertain.

**Case Report:**

We report the case of a woman in her 70s, who was previously tracheostomized at another facility and was presented to our attention with glottic-subglottic stenosis. We performed a lysis of glottic synechia and subglottic debulking via transoral laser microsurgery, yielding satisfactory results over the short term. However, a relapse occurred within two months, along with ulcerative lesions on the nasal septum. Biopsies revealed multinucleated giant cells and inflammation suggestive of vasculitis. Based on the histological and clinical features, a diagnosis of vasculitis was considered. Anti-Neutrophil Cytoplasmic Antibodies testing was positive. A rheumatological examination confirmed the hypothesis of Granulomatosis with Polyangiitis. The lack of typical symptoms was the main reason for the delayed diagnosis.

**Conclusion:**

Involvement of the subglottic region and the upper portion of the trachea is a rare but severe complication of GPA. The current literature reports only few cases of laryngeal stenosis, with poor prognosis. Histological examinations of biopsied laryngeal tissue showed significant but non-specific inflammation, contributing to the delay in diagnosis. There are still no precise guidelines for the surgical treatment of subglottic stenosis. This case underscores the importance of considering laryngeal involvement in GPA for early diagnosis and timely intervention to prevent serious complications in order to improve patient outcomes.

## Introduction

Granulomatosis with Polyangiitis (GPA), also known as Wegener’s Granulomatosis, is an ANCA (antineutrophil cytoplasmic antibodies)-associated vasculitis that primarily targets small vessels, leading to necrotizing granulomatous reactions in the airways and small vessels, as well as necrotizing glomerulonephritis (1). ANCAs are autoantibodies directed against some constituents of azurophil granules of neutrophils, particularly Myeloperoxidase (MPO) and Proteinase-3 (PR-3) (2). The prevalence of GPA is estimated at 1 in 6,400 to 1 in 42,000 cases, with higher incidence in colder regions and among Caucasian populations. The average age of onset is 45 years, although it can occur at any age, affecting both males and females equally. The etiology remains uncertain, but genetic susceptibility, environmental agents, infectious episodes, and abnormalities in innate and adaptive immunity may be involved (1).

We report the case of a 70-year-old woman with glottic-subglottic stenosis, who required an emergency tracheostomy due to undiagnosed GPA. This case is presented in accordance with the SCARE criteria (3).

## Case Report

**Fig 1:**
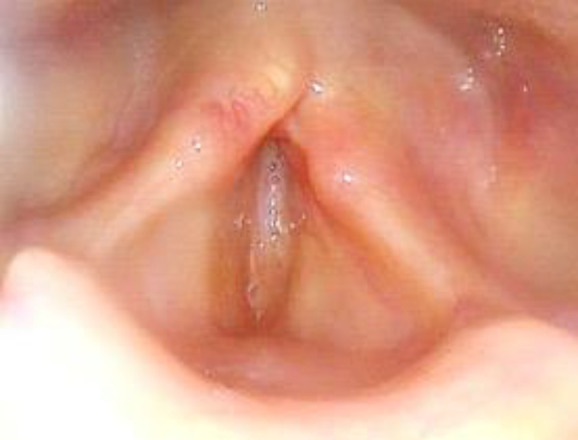
Laryngoscopy image showing glottic stenosis with complete adduction of true vocal cords.

A Caucasian woman in her 70s, who had previously undergone a tracheostomy during an emergency respiratory crisis of unknown cause in 2018 at another hospital, presented to our ENT Unit for evaluation of tracheostomy closure. Fiberoptic nasopharyngolaryngoscopy revealed glottic adherence of the true vocal cords along the entire free edge (Figure 1), with preserved but limited arytenoid motility due to synechia. 

**Fig 2:**
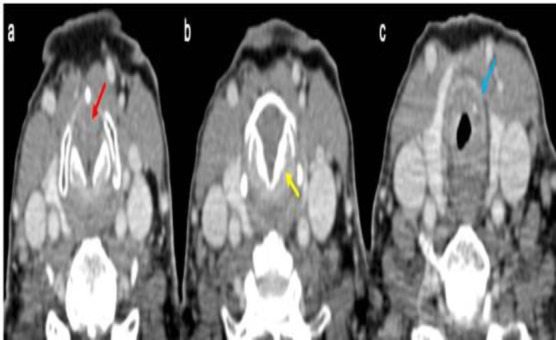
CT images of glottic-subglottic stenosis: (a) glottic plane (red arrow), (b) subglottic plane showing cricoid cartilage thickening (yellow arrow), (c) first tracheal ring (blue arrow).

A neck computed tomography (CT) showed asymmetric thickening of the lamina of cricoid cartilage and narrowing of the posterior subglottic space (Figure 2). 

**Fig 3:**
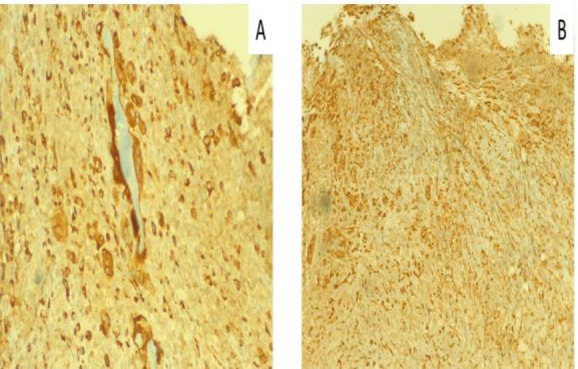
Laryngeal (A) and nasal (B) mucosa showing CD68+ multinucleated giant cells (granulomas).

To restore laryngeal breathing and remove the tracheostomy, we performed a lysis of glottic synechia and subglottic debulking using transoral laser microsurgery (TLM). Histological analysis of the laryngeal tissue revealed intense lymphoplasmacytic, histiocytic, and granulocytic inflammation with perivascular distribution and multinucleated giant cells (Figure 3). 

**Fig 4:**
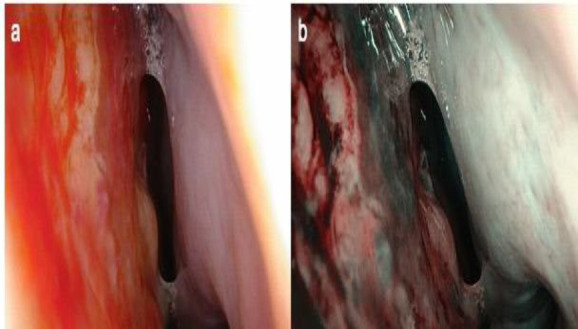
Ulcerative lesions in the naso-septal mucosa on white light (a) and NBI (b) endoscopy.

After the surgery, the patient’s normal airway function was temporarily restored. However, two months later, follow-up showed a relapse of laryngeal synechiae and ulcerative lesions in the nasal septum (Figure 4). Nasal biopsies confirmed intense lympho-granulocytic inflammation with multinucleated giant cells. Based on the histological and clinical features, a diagnosis of vasculitis was considered. The ANCA blood testing came back as positive. 

Therefore, considering the histological examinations (multinucleated giant cells and perivascular inflammation), the progressive nasal deformity (into a saddle-shaped form), and the ANCA positivity, a rheumatology consultation confirmed the diagnosis of Granulomatosis with Polyangiitis, predominantly localized to the larynx. The patient was then started on methotrexate therapy, with definitive laryngeal surgery postponed due to a subsequent diagnosis of breast cancer.

## Discussion

GPA is a small vessel vasculitis classified as an ANCA-associated vasculitis. It is characterized by necrotizing granulomatous inflammation of the respiratory tract, necrotizing vasculitis of small and medium-sized vessels and frequent necrotizing glomerulonephritis. It is a multi-organ disease, primarily affecting the lungs, kidneys, the head and neck regions. Clinical signs in the ear, nose, and throat occur in 50-95% of patients, while bronchopulmonary and/or renal symptoms are present in 60-80%. Kuhn et al. evaluated ENT symptoms in 230 patients with GPA and found that 59% had nasal obstruction, 57% had anosmia, 23% had hearing loss, and only 15% had laryngeal involvement (4-6). Involvement of the subglottic region and the upper portion of the trachea is a rare but severe complication of GPA. Current literature reports only a few cases of laryngeal stenosis with poor prognosis (7-10). Moreover, studies indicate that subglottic stenosis in GPA primarily affects younger individuals (under 20 years of age) and may develop even outside active disease phases (7-8). In our case report, the patient was an elderly woman (70 years old) with no prior history of significant pathology, who underwent emergency tracheostomy due to severe inspiratory dyspnea, without the prodromal symptoms (such as hoarseness, stridor, etc.). Histological examinations of the biopsied laryngeal tissue revealed significant but non-specific inflammation, leading to a delay in diagnosis. Regarding surgical treatment of subglottic stenosis, no specific guidelines currently exist. Feinstein et al. categorized surgical techniques into open and endoscopic (11). The latter includes rigid or balloon dilation, radial incisions using TLM or cold knife, and the placement of endotracheal stents. Later, topical administration of mitomycin and/or glucocorticoids can be performed. In their study, Feinstein et al. (9) found no significant difference between these surgical techniques in terms of the interval free from recurrence of stenosis. However, they did note that topical administration of mitomycin can extend the recurrence-free interval. Wierzbicka et al. suggested that endotracheal stents should be reserved for extremely selected cases, as they can act as foreign bodies, promoting granulation of the airway mucosa and further narrowing the lumen (12).

## Conclusion

This case highlights the critical importance of early recognition and treatment of laryngeal involvement in GPA, given its rarity and its potential for severe complications. Despite the typical multi-organ involvement in GPA, its presentation can be atypical and insidious, as demonstrated by this elderly patient who lacked classic symptoms but suffered significant airway compromise nevertheless. The delayed diagnosis due to non-specific inflammatory histological findings emphasizes the necessity for heightened clinical suspicion and comprehensive evaluation in similar scenarios. Early identification and intervention are paramount to prevent life-threatening complications such as airway obstruction and to improve the patient’s quality of life and long-term prognosis. This case also draws attention to the need for interdisciplinary collaboration involving ENT specialists, rheumatologists, and pathologists to ensure accurate diagnosis and effective management. Furthermore, this case report contributes to the limited literature on GPA, advocating for greater awareness of its potential presentation with laryngeal manifestations.

## Note

Written informed consent was obtained from the patient for the publication of this case report.
